# Mental health and COVID-19 in a general population cohort in Spain (COVICAT study)

**DOI:** 10.1007/s00127-022-02303-0

**Published:** 2022-05-28

**Authors:** X. Goldberg, G. Castaño-Vinyals, A. Espinosa, A. Carreras, L. Liutsko, E. Sicuri, M. Foraster, C. O’Callaghan-Gordo, P. Dadvand, G. Moncunill, C. Dobaño, B. Cortés, V. Pleguezuelos, K. Straif, J. Garcia-Aymerich, R. de Cid, E. Cardis, M. Kogevinas

**Affiliations:** 1 ISGlobal, Barcelona, Spain, Barcelona, Spain; 2 Mental Health Department, Institut d’Investigació I Innovació Parc Taulí I3PT, Sabadell, Spain; 3 CIBER Salud Mental (CIBERSAM), Madrid, Spain; 4 IMIM (Hospital del Mar Medical Research Institute), Barcelona, Spain; 5 Universitat Pompeu Fabra (UPF), Barcelona, Spain; 6 CIBER Epidemiología Y Salud Pública (CIBERESP), Madrid, Spain; 7 Genomes for Life-GCAT Lab, Germans Trias I Pujol Research Institute (IGTP), Badalona, Spain; 8 UrFU, Yekaterinburg, Russia; 9 Universitat Oberta de Catalunya, Barcelona, Spain; 10 Banc de Sang I Teixits (BST), Barcelona, Spain; 11 Boston College, Chestnut Hill, MA USA; 12 PHAGEX Research Group, Universitat Ramon Llull, Blanquerna School of Health Science, Barcelona, Spain; 13 ISGlobal, Hospital Clínic - Universitat de Barcelona, Barcelona, Spain

**Keywords:** Anxiety, Depression, COVID-19, Lockdown, Cohort study

## Abstract

**Purpose:**

Mental health conditions may affect outcome of COVID-19 disease, while exposure to stressors during the pandemic may impact mental health. The purpose of this study was to examine these factors in relation to ocurrence of depression and anxiety after the first outbreak in Spain.

**Methods:**

We contacted 9515 participants from a population-based cohort study in Catalonia between May and October 2020. We drew blood samples to establish infection to the virus. Pre-pandemic mental health conditions were confirmed through Electronic Health Registries. We used the Hospital Anxiety and Depression Scale to assess severe depression and anxiety post-pandemic. Exposure to proximal, financial and wider environment stressors during the lockdown were collected. We calculated Relative Risks (RR), adjusting for individual- and contextual covariates.

**Results:**

Pre-pandemic mental health disorders were not associated with SARS-CoV-2 infection , but were associated with severity of COVID-19 disease. People with pre-existing mental health disorders showed higher prevalence of severe depression (25.4%) and anxiety (37.8%) than those without prior mental disorders (4.9% and 10.1%). Living alone was a strong predictor of severe depression among mental health patients (RR = 1.6, 95% CI 1.2–2.2). Among those without prior mental health disorders, post-lockdown depression and anxiety were associated with household interpersonal conflicts (RR = 2.6, 95% CI 2.1–3.1; RR = 2.1, 95% CI 1.9–2.4) and financial instability (RR = 2.2, 95% CI 1.8–2.9; 1.9, 95% CI 1.6–2.2).

**Conclusions:**

The COVID-19 pandemic and the lockdown were associated with increased post-lockdown depression and anxiety. Patients with pre-existing mental health conditions are a vulnerable group for severe COVID-19 disease.

**Supplementary Information:**

The online version contains supplementary material available at 10.1007/s00127-022-02303-0.

## Introduction

From the beginning of the coronavirus disease 2019 (COVID-19) pandemic, higher rates of severe depression and anxiety were expected in association with the outbreak itself (e.g. fear of getting infected) and with stressors originating from the disruptions in living and socioeconomic conditions following the stay-at-home orders [1, 2]. These expectations were promoted by previous evidence on vulnerable groups showing that enforced isolation, household interpersonal conflicts, job insecurity, and environmental hazards are stressors associated with clinically relevant depression and anxiety [3–5]. After the outbreak of the COVID-19 disease, population-based studies provided initial confirmation to these associations [6].

The effects of potential stressors in different population groups are still unclear. A key component to understand these relationships are inequalities that can potentially shape the rates of the disorders and the risks associated with each of the stressors. For example, women survivors of domestic violence have a two-time increased risk of depression and anxiety [7], but the impact of this exposure may be different in the context of the confinement during the pandemic. Similarly, stressors such as isolation may have been particularly hard in some age groups, potentially modifying the age-dependent rates of depression and anxiety. Therefore, the examination of the effects of the pandemic response measures on mental health requires disaggregating the results according to these factors that may underlie an unequal distribution of susceptibility [8].

Patients with prior mental health conditions have been found to be more prone to COVID-19 disease [9]. They may have a reduced awareness of risk that can lead to increased exposure to hazards and more barriers in accessing adequate treatment [10]. Pathophysiological features involving the hypothalamic–pituitary–adrenal (HPA) axis, autonomic nervous system and immunological response may increase the patients’ neurobiological vulnerability to the disease [11, 12]. Recent analyses have identified gene clusters and inflammatory signalling pathways shared between COVID-19 and mental disorders that suggest common pathogenic mechanisms [13, 14]. In turn, people with pre-existing depression and anxiety were expected to present a worsening of their mental health status after the first outbreak of the COVID-19 pandemic. Being a vulnerable group, they may be differentially affected by risk factors such as unemployment, isolation, and other stressors that were frequent in the context of the confinement [15].

Spain was among the hardest-hit countries in Europe during the first months of the outbreak reaching almost 10,000 cases and over 1000 deaths per day in early April 2020. This led to firm restrictions in a society strongly defined by family ties and outdoor life, and an economy largely dependent on tourism and restaurant business. A stay-at home order was issued at the national level starting March 15th and lasted until April 26th. Official registers showed a 21% increase in the rates of unemployment in May 2020 relative to May 2019 [16]. Calls to emergency numbers reporting domestic violence increased by 80% in May 2020 relative to February 2020 [17].

In the present study, we examined in a large population-based cohort in northern Spain (Catalonia) the association of pre-pandemic mental conditions with infection to SARS-CoV-2 and severity of COVID-19 disease. We describe the prevalence of severe depression and anxiety following the first wave of the COVID-19 pandemic and explore the association between proximal, financial and wider environment stressors during the lockdown with the prevalence of depression and anxiety, disaggregated by previous history of mental health diagnoses. We finally examine these factors across vulnerable groups defined by age, gender, and socioeconomic status (SES).

## Methods

### Study design and participants

The COVICAT Study (COVID-19 cohort in Catalonia) is a prospective epidemiological study that aims to describe the health impact of the COVID-19 pandemic on the adult population in northern Spain. It builds on pre-existing cohort studies that were established before the outbreak. A single harmonized protocol was used for all five cohorts included in the COVICAT study that comprised all living cohort participants with an email address or telephone number. 88.5% of the COVICAT study where participants of a single cohort (GCAT) [18]. The description of the individual cohort studies, references, and inclusion criteria are described in Supplementary materials pp.2–3. After the first wave of the COVID-19 pandemic in Spain in March 2020, we harmonized data of all cohorts and participants were contacted. They responded to a questionnaire and draw blood samples to determine SARS-CoV-2 seroprevalence. Data collection was primarily completed on a study portal website, while we conducted telephone interviews for participants unfamiliar with web-based approaches (*n* = 577, 6.1%). All participants provided informed consent, and we obtained ethical approval for the study from the Parc de Salut Mar Ethics Committee (CEIm-PS MAR, number 2020/9307/I).

We contacted most participants in our study (99.7%) between May 28th and August 15th, 2020, coincident with the progression towards the “new normality.” This phase was characterized by increased mobility, opening of commercial activities at 50% space capacity, and priority times for outings of vulnerable groups. These restrictions were progressively reduced until the new normality was reached in mid-June. Out of the eligible participants who were contacted 10,862 (61.5%) agreed to participate; of these, 10,087 (92.9%) participants completed the interview satisfactorily. In the present report, we excluded 34 participants who were interviewed in autumn (between October and November, 2020), when new restrictions were applied. Pre-pandemic information of diagnosis of mental health disorder could not be confirmed through Electronic Health Records in 538 participants, and these cases were excluded from the analysis. Complete records were available for 9515 participants.

### Measures

The study primary outcomes were severe depression and anxiety experienced by the participants at the time of data collection. These outcomes were assessed using the Hospital Anxiety and Depression Scale (HADS) [19], which provided a measure of ongoing symptoms of anxiety and depression through 14 items scored on a four-point Likert-type scale. The HADS can be used for diagnostic purposes and for assessing the severity of the disorders through the Depression and Anxiety subscales. Each subscale counts 7 items and ranges between 0 and 21. Following the cut-off points validated in the Spanish population [20], we used a score of 11 for each subscale to identify both severe anxiety and severe depression, while moderate levels of anxiety were settled at 8 and moderate levels of depression at 6. Severe anxiety and depression levels indicate clinically relevant diagnosis [21]).

Participants were asked whether they had ever been diagnosed by a doctor with depression, anxiety or other mental health illness. Lifetime self-reported diagnoses were confirmed through Electronic Health Records of the Public Healthcare System linked to each participant through the Health Personal Identification Number. This system provides detailed data on mental health diagnoses according to the International Classification of Diseases, 9^th^ revision, registered by healthcare professionals in clinical settings since 2012.

We identified the stressors of interest during confinement (March 15th to April 26th, 2020) among factors previously proposed to have a disrupting effect [1] and conceptualized them as (1) proximal, (2) financial, (3) wider environment factors according to the established ecological systems theory [22] and the social determinants of mental health model [5]. We also evaluated COVID-19 disease as a potential stressor:

1. Proximal factors.
  1.1 The number of people living in the household during the confinement was used as an indicator of isolation. Living alone, in contrast to sharing the household with 1 or more people, was considered of risk for depression/anxiety.
  1.2 Media exposure during the confinement was examined through the frequency of use of media to check information about the pandemic, ranked several times a day, daily, weekly or more. Consulting information several times a day was considered high media exposure.
  1.3 Data on interpersonal conflicts in the household during the confinement were collected by asking participants to state indicate the degree of agreement with a statement that they had difficulties to cope with lockdown due to conflicts with other household members. A complete or partial agreement was considered of risk.
  1.4 Caregiving activities during the confinement were explored by asking participants whether they were in charge of caregiving for children in contrast to their partner, a third party or shared responsibilities within the adults in the household. Being in charge of caregiving activities was considered of risk.
2. Financial factors.
  2.1 Concerns regarding financial instability were explored through the question: “As a consequence of the lockdown, are you in a situation where you cannot face common expenses such as rent or food?”. A positive response was considered of risk and captured as “Struggle to pay rent/food”.
  2.2 Employment status at the time of assessment was collected and unemployment during the lockdown was analysed compared to the rest of status (employed, others including retired, student, and household work).
3. Wider environment factors.
  3.1 Access to outdoor facilities during confinement was explored by rating the availability and frequency of use of any balcony, terrace or garden in this period, and dichotomizing the answers to code for frequent/very frequent access versus rare/no access (risk).
  3.2 Participants had to rate how much they were annoyed by noise coming from outside their homes (traffic, essential commercial and industrial, neighbours) during confinement using a 11-point Likert scale. This variable was dichotomized as no/low annoyance (score 0–5) *versus* moderate/high annoyance (score 6–10 indicating risk).
4. COVID-19.

Participants were asked about a positive test for SARS-CoV-2 infection, COVID-19 hospitalization, presence of COVID-19 symptoms or contact with diagnosed COVID-19 case. Based on this information, we defined 469 cases of COVID-19 [23]. Severe COVID cases were those reporting COVID-19 hospital or Intensive Care Unit (ICU) admission (*n* = 59) verified through electronic records.

The survey’s digitalized format forced the users to respond to all items of the scales before submitting, which prevented missing items as well as missing HADS scores. Two predictors used in this study (media exposure and access to outdoor spaces) were omitted in the telephone survey due to the length of the interview and those participants are not included in analyses of these variables. The total number of participants for each analysis is reported in the table footnotes.

All participants were invited to participate in the serological study and 8906 agreed. We collected blood samples from 4103 participants randomly selected from those agreeing to participate. The assay used measured levels of IgM, IgA and IgG to five SARS-CoV-2 antigens and defined seropositivity using pre-pandemic control samples. Assay performance has been described elsewhere [24]. We detected SARS-CoV-2 antibodies in 70% of self-reported cases and in 90% of participants reporting prior COVID-19 hospital admission. [23].

Data on age, gender and educational level were collected. Educational level was used as a proxy for socioeconomic status: higher education level (graduate or above) indexed higher socioeconomic status. Number of days passed from end of stay-at-home order to the interview date was recorded and explored to control for potential differences in emotional states related to the different phases of the deconfinement process.

### Statistical analysis

We applied log-binomial regression models to estimate Relative Risks (RR) and 95% CIs for the association between the stressors and the outcomes, adjusted for age, gender, education level, days passed from end of stay-at-home order to the interview date, type of interview (online/telephone), pre-pandemic diagnosis of mental health (MH) disorder and (when appropriate) COVID-19 disease. When convergence was not achieved, we applied Poisson regression models with robust standard errors. We examined potential differences in the associations across vulnerable groups. Effect modification by gender, age, SES and pre-pandemic diagnosis of mental health disorder was assessed by including the interaction term in the models. Relative Risks for each stratum were derived from the interaction model. We also applied log-binomial regression to estimate Relative Risks (RR) and 95% CIs for models evaluating COVID-19 outcomes (SAR-CoV-2 infection, COVID-19 and severity). All statistical analyses were performed using Stata S.E. version 16 (StataCorp LLC, Texas, USA).

To extrapolate the prevalence of severe depression and anxiety to the overall population, we calibrated study data to generate estimates representing the population of the region aged more than 20 years from the 2019 national census (INE 2019), including age, sex, education, smoking, and health region. We calculated sampling weights using iterative proportional fitting or "raking" [25] to balance the study sample characteristics to those of the population. The COVICAT study includes lower numbers of younger ages (less than 40), which leads to overdispersed weights. For this reason, we restricted extreme weights by trimming the distribution at 99% of the weights.

## Results

The mean age of the participants was 54.6 years old (age range 20–72) with a slightly higher proportion of women and 46% with education level graduate or above (Table 1). The mean number of days passed between the end of the stay-at-home order and the date of the interview was 45.8 days. Almost 5% of the sample had a diagnosis of COVID-19 disease (*N* = 469). Table 1 shows the frequencies of confirmed pre-pandemic depression (*n* = 225; diagnosis of major depressive disorder = 87 cases, dysthymia = 155 cases) and anxiety (*n* = 267; diagnosis of anxiety disorder = 248 cases, phobias = 20 cases, obsessive–compulsive disorder = 17 cases). Other diagnoses were: bipolar disorder Type I (21 cases), schizophrenia (8 cases), and eating disorders (19 cases). Participants with pre-pandemic diagnosis of mental health disorder were more frequently women (70.7%) in their 50’s (51.9%) and presented a secondary-level education (46.2%).

**Table 1:** Description of sociodemographic characteristics and distribution of the proximal, financial and wider environment factors (*n* = 9515), COVICAT study

	*n* (%)
Gender	
Women	5668 (59.6)
Men	3847 (40.4)
Age groups	
≤ 49	2591 (27.2)
50–59	4386 (46.1)
≥ 60	2538 (26.7)
Education	
Primary or lower	1082 (11.4)
Secondary	4021 (42.3)
Graduate and above	4412 (46.4)
COVID-19 disease	
All cases	469 (4.9)
Severe COVID-19	59 (0.6)
No diagnosis	9046 (95.1)
Pre-pandemic mental health diagnosis	
Any diagnosis	563 (5.9)
Depression	225 (2.4)
Anxiety	267 (2.8)
No diagnosis	8952 (94.1)
Time from end of confinement to interview	
30 to 45 days	7189 (75.6)
46 days to 2 months	470 (4.9)
2 to 3 months	1285 (13.5)
3 to 4 months	571 (6.0)
Number of people in household	
Living alone	1291 (13.6)
Two persons	2973 (31.2)
Three persons	2479 (26.1)
Four or more people	2772 (29.1)
Media exposure	
Several times a day	2320 (24.4)
Daily	4231 (44.5)
Weekly or less	2879 (30.3)
Unknown	85 (0.9)
Interpersonal conflicts	
Important difficulties	902 (9.5)
Moderate difficulties	1738 (18.3)
No difficulties	6875 (72.3)
Caregiving of children	
Yes	1804 (19.0)
Shared responsibility	2516 (26.4)
Another persons' responsibility	497 (5.2)
No dependent children	4698 (49.4)
Employment status	
Currently unemployed	926 (9.7)
Currently employed	6213 (65.3)
Others	2376 (25)
Struggle to pay rent/food	
Yes	814 (8.6)
No	8701 (91.4)
Access to outdoor spaces	
No access or only rare access	2117 (22.2)
Some access	3834 (40.3)
Often access	3479 (36.6)
Unknown	85 (0.9)
Noise annoyance	
High levels	2871 (30.2)
Low level of annoyance	3715 (39.0)
No annoyance	2929 (30.8)

## Pre-pandemic mental health diagnosis, SARS-CoV-2 infection and COVID-19

We examined whether the risk of SARS-CoV-2 infection (measured through antibody levels), COVID-19 disease and severity of the disease were associated with having pre-pandemic diagnosed mental health conditions compared to those not diagnosed with these conditions (Table 2). The risk of SARS-CoV-2 infection was explored among participants with serological testing (*N* = 3879). There was no evidence of association between prior mental health diagnosis and infection (RR = 1.17, 95% CI = 0.89–1.54 for any prior disease). Compared to participants with no prior mental health diagnosis, patients had an increased risk of COVID-19 disease (RR = 1.64, 95% CI = 1.22–2.21 for any prior mental health diagnosis). Risk was higher for those with severe COVID-19 (hospitalized or ICU) (RR = 3.60, 95% CI = 1.83–7.10). Patients diagnosed with depression tended to have higher risks than those diagnosed with anxiety (Table 2).

**Table 2:** Risk of SARS-CoV-2 infection, COVID-19 disease and COVID-19 severity among patients with confirmed pre-pandemic diagnosis of mental health disorder compared to those without a pre-pandemic mental health diagnosis, COVICAT study

	SARS-CoV-2 infection	COVID-19 disease	COVID-19 disease severity	
			Mild cases vs controls	Severe cases vs controls
	RR (95% CI)	RR (95% CI)	RR (95% CI)	RR (95% CI)
Pre-pandemic diagnosis of depression	1.16 (0.76–1.76)	2.39 (1.66–3.46)	2.10 (1.38–3.20)	5.90 (2.54–13.69)
Pre-pandemic diagnosis of anxiety	1.15 (0.75–1.75)	1.76 (1.19–2.62)	1.58 (1.01–2.47)	3.62 (1.45–9.04)
Any Pre-pandemic mental health diagnosis	1.17 (0.89– 1.54)	1.64 (1.22–2.21)	1.44 (1.03–2.01)	3.60 (1.83–7.10)

Relative Risks (RR) and 95%CI from log-binomial regression models adjusted for age, sex, education level and type of survey

Analyses were run among participants with available information for all variables; sample sizes were *N* = 9515 for COVID-19 disease and *N* = 3879 for serology testing (SARS-CoV-2 infection)

## Post-lockdown prevalence of anxiety and depression

The prevalence of severe depression at the COVICAT study after the outbreak (measured with HADS) was 5.9% and of severe anxiety 11.8% (Table 3). Prevalence was higher among participants with any pre-pandemic mental health disorders (25.4% for severe depression and 37.8% for severe anxiety) compared to those without pre-pandemic diagnosis (4.9% severe depression, 10.1% severe anxiety). Women, people under the age of 50, and those in low-and middle-SES, showed higher prevalence than men, those aged 50 and above and those in high SES (Table 3).

**Table 3:** Prevalence of post-lockdown moderate/severe depression and anxiety according to pre-pandemic diagnosis of mental health disorder and sociodemographic characteristics, COVICAT study, *n* = 9515

	Depression			Anxiety		
	None	Moderate	Severe	None	Moderate	Severe
	*n* (%)	*n* (%)	*n* (%)	*n* (%)	*n* (%)	*n* (%)
Total	6916 (72.7)	2037 (21.4)	562 (5.9)	6580 (69.2)	1815 (19.1)	1120 (11.8)
Pre-pandemic mental health diagnosis						
No diagnosis	6683 (74.6)	1850 (20.7)	419 (4.9)	6378 (71.3)	1667 (18.6)	907 (10.1)
Any diagnosis	233 (41.4)	187 (33.2)	143 (25.4)	202 (35.9)	148 (26.3)	213 (37.8)
Depression	89 (39.6)	70 (31.1)	66 (29.3)	84 (37.3)	56 (24.9)	85 (37.8)
Anxiety	103 (38.6)	88 (32.9)	76 (28.5)	79 (29.6)	70 (26.2)	118 (44.2)
Gender						
Women	3907 (68.9)	1352 (23.9)	409 (7.2)	3586 (63.3)	1243 (21.9)	839 (14.8)
Men	3009 (78.2)	685 (17.8)	153 (3.9)	2994 (77.8)	572 (14.9)	281 (7.3)
Age groups						
≤ 49	1801 (69.5)	589 (22.7)	201 (7.8)	1700 (65.6)	490 (18.9)	401 (15.5)
50–59	3107 (70.1)	1003 (22.8)	276 (6.3)	2953 (67.3)	880 (20.1)	553 (12.6)
≥ 60	2008 (79.1)	445 (17.5)	85 (3.3)	1927 (75.9)	445 (17.5)	166 (6.5)
Education						
Primary or lower	759 (70.2)	243 (22.5)	80 (7.4)	708 (65.4)	228 (21.1)	146 (13.5)
Secondary	2859 (71.7)	898 (22.3)	264 (6.6)	2701 (67.2)	783 (19.5)	537 (13.3)
Graduate or above	3298 (74.8)	896 (20.3)	218 (4.9)	3171 (71.8)	804 (18.2)	437 (9.9)
COVID-19 disease						
All cases	289 (61.6)	133 (28.4)	47 (10.0)	269 (57.4)	110 (23.5)	90 (19.2)
Severe COVID-19*	38 (64.4)	14 (23.7)	7 (11.9)	32 (54.2)	16 (27.1)	11 (18.6)
No diagnosis	6627 (73.3)	1904 (21.1)	515 (5.7)	6311 (69.8)	1705 (18.9)	1030 (11.4)

*Severe cases among those with positive COVID-19 diagnosis (*N* = 469)

We extrapolated these estimates to the total adult population of Catalonia, using weights calculated based on sex, age, education and area of Catalonia distribution in the adult population. The post-pandemic prevalence in the total adult population of Catalonia was estimated to be 5.0% (*n* = 305,207) for severe depression and 10.7% for severe anxiety (*n* = 653,229 cases).

## Stressors during lockdown, anxiety and depression

We explored the association between stressors during lockdown and severity of post-lockdown depression and anxiety (Table 4). All stressors (with the exception of living alone for severe anxiety) were associated with severe depression and severe anxiety. The highest RR were observed for household interpersonal conflicts in relation to severe depression (RR = 2.22, 95% CI = 1.90–2-59) and severe anxiety (RR = 1.97, 95% CI = 1.77–2.19). There was also a strong effect of financial factors, specifically struggle to pay for rent/food (RR = 2.05, 95% CI = 1.70–2.47 for severe depression; RR = 1.77, 95% CI = 1.55–2.01 for severe anxiety) and also for unemployment in relation to severe depression (RR = 1.81, 95% CI = 1.49–2.20). Having been diagnosed with COVID-19 disease was associated with both severe depression and severe anxiety (Table 4).

**Table 4:** Association between stressors during the lockdown and COVID-19 disease, with post-lockdown severity of depression

	Depression		Anxiety	
	Moderate (20.3%)	Severe (4.5%)	Moderate (18.4%)	Severe (9.9%)
	RR (95% CI)	RR (95% CI)	RR (95% CI)	RR (95% CI)
Household conditions				
Living alone	1.07 (0.96–1.19)	1.35 (1.10–1.65)	0.95 (0.85–1.07)	0.94 (0.80–1.10)
High media exposure (**)	1.32 (1.21–1.43)	1.56 (1.32–1.84)	1.32 (1.21–1.44)	1.60 (1.43–1.78)
Interpersonal conflicts	1.71 (1.59–1.85)	2.22 (1.90–2.59)	1.61 (1.48–1.74)	1.97 (1.77–2.19)
Caregiving of children	1.09 (0.99–1.20)	1.18 (0.99–1.40)	1.15 (1.04–1.26)	1.14 (1.01–1.28)
Financial strain				
Currently unemployed	1.17 (1.04–1.32)	1.81 (1.49–2.20)	1.24 (1.09–1.40)	1.33 (1.15–1.53)
Struggle to pay rent/food	1.16 (1.02–1.31)	2.05 (1.70–2.47)	1.27 (1.11–1.45)	1.77 (1.55–2.01)
Wider environment				
Rare/no access to outdoor spaces (**)	1.09 (1.00–1.19)	1.49 (1.26–1.76)	1.01 (0.91–1.11)	1.25 (1.11–1.40)
Noise annoyance	1.34 (1.24–1.45)	1.43 (1.23–1.68)	1.27 (1.17–1.38)	1.42 (1.27–1.57)
COVID-19 (***)				
All cases	1.32 (1.14–1.53)	1.56 (1.19–2.05)	1.29 (1.09–1.51)	1.49 (1.24–1.79)

Log-binomial/Poisson regression analysis*, COVICAT study, *n* = 9515 **

*Log-binomial regression models adjusted for age, gender, education level, days passed since end of stay-at-home order, positive COVID-19 diagnosis, confirmed history of mental health diagnosis, and type of interview. Poisson regression models with robust standard errors were applied when convergence was not achieved

**Analyses were run among participants with available information for all variables; sample sizes were *N* = 9515 for all models except those marked with (**), which were *N* = 9430 due to the omission of these questions in the telephone-based interviews

***Model for COVID-19 adjusted as above except for positive COVID-19 diagnosis

**Fig. 1:**
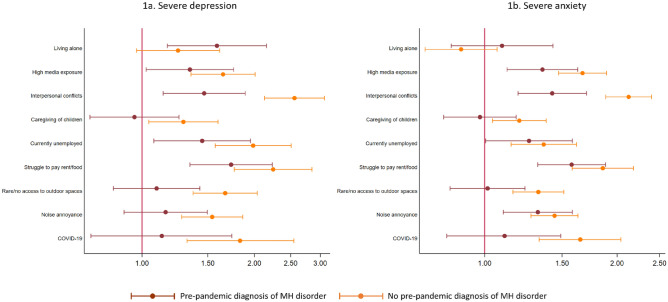
Associations between exposures and severe depression (**a**) and anxiety (**b**) by pre-pandemic diagnosis of mental health (MH) disorder from log-binomial/Poisson regressions. Dots indicate estimates of relative risks and bars represent 95% CI. Analyses were adjusted for gender, age, education level, days passed since end of stay-at-home order, COVID-19 diagnosis, and type of interview

When stratifying by pre-pandemic diagnosis of mental health disorders, patterns of risk of severe depression and anxiety in relation to stressors tended to be lower among those with prior mental health disorders as compared to risks found among persons without prior mental health disorders (Fig. 1). The only exception was the association between living alone and severe depression and anxiety, which was higher in the group of mental health patients (Fig. 1 and Supplementary Table S1 for numerical estimates).

**Fig. 2:**
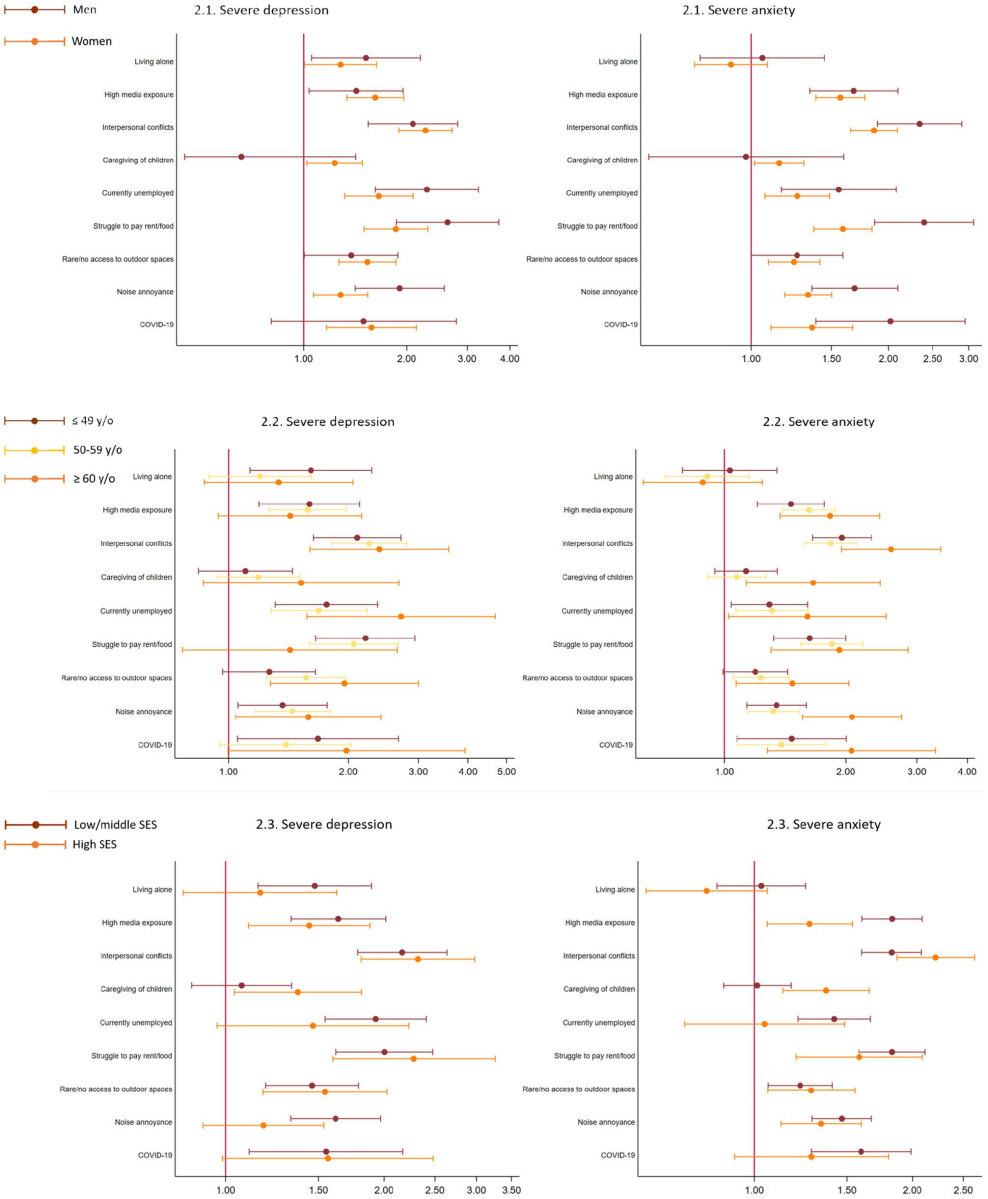
Associations between exposures and severe depression and anxiety by (1) gender, (2) age group, (3) socioeconomic status (SES) from log-binomial/Poisson regressions. Dots indicate estimates of relative risks and bars represent 95%CI

The impact of unemployment and struggle to pay for rent/food was larger among men, in particular for anxiety (interaction *p* < 0.01). Women showed higher increases in RR of depression in association with noise annoyance (interaction *p* < 0.05). The widest difference by age was found for noise annoyance with a higher RR of anxiety among persons above 60 (interaction *p* < 0.05). Finally, caregiving of children was associated with higher RR of anxiety in high SES group compared to low/middle SES group (interaction *p* < 0.05) (Fig. 2 and Supplementary Tables S2-S4).

## Discussion

Using a large population-based cohort of adult participants in northern Spain, we found that diagnosed pre-pandemic mental health disorders were associated with a higher risk of severe COVID-19 disease although not for a higher risk of infection by SARS-CoV-2. 5.7% of people presented severe depression and 11.5% severe anxiety after the first wave of the COVID-19 pandemic and the prevalence was four- to five-times higher among those with confirmed history of mental health diagnosis. Post-lockdown increased risks of anxiety and depression were associated with proximal, financial and environmental stressors experienced during the lockdown and with COVID-19 disease, and were dependent upon pre-pandemic social, economic and health inequalities.

The finding that patients with previous history of mental health treatment presented high prevalence of depression and anxiety during the lockdown has been proposed in studies using self-reported measures as perceived by the patients [26, 27], case–control reports measuring symptoms through scales [28] and longitudinal population-based studies [15]. However, the mechanisms underlying this increased risk of severe symptomatology are not fully understood. We found that the associations between the increases in prevalence of depression and anxiety and the stressors were in general weaker among patients when compared to the group of participants without pre-existing mental illness. The only exception was living alone, which was associated with a RR = 1.59 among these patients (95% CI = 1.17–2.15). This finding supports a role for forced physical distancing as a potential detrimental influence among patients with a history of mental health diagnosis. The emotional state of loneliness has been found to predict mental health conditions [29], and it could be the case that those living alone may experience this emotional state more frequently and/or more severely than those sharing the household. Other factors not included in our study, such as disruption of healthcare and reduced access to medication, may have had a large influence in the mental state of people requiring treatment as was the case for other patients with chronic disorders [30–32]. Further research is needed in longitudinal follow-ups of patients using pre-pandemic mental health registries and treatment disruptions.

We found that patients with prior mental health conditions were more frequently affected by COVID-19 disease, and the presentation of the disease was more severe. In turn, having a diagnosis of COVID-19 was associated with increased risk of presenting depression and anxiety during the confinement. This bi-directional relationship between mental health and COVID-19 is supported by most, although not all, cohort studies based on pre-pandemic records [33–35] and reports of patients with long-COVID [36]. Potential explanations include psychosocial characteristics involving behavioral and lifestyle patterns along with a high symptoms awareness and close access to the health system that could have facilitated the diagnosis. Importantly, shared biological mechanisms affecting brain areas implicated in mood regulation or related to psychopharmacological treatment may explain the findings. We did not find evidence for higher seropositivity in the group of patients, indicating that previous mental health conditions do not increase susceptibility to infection with SARS-CoV-2. Among those infected, however, prior mental health conditions did predispose to more severe COVID-19 disease. Most reports focusing on people with previous mental disorders did not include information on antibodies levels. Interestingly, a study exploring severe mental disorders (i.e. schizophrenia, bipolar disorder) found that risk of SARS-CoV-2 seropositivity was lower in patients [37]. Altered neuroendocrine regulation and associated interactions with the immune system are key features of depression and anxiety. COVID-19 disease impacts similar pathways and shows significant genetic correlations with mental disorders [38]. Increased severity of COVID-19 among people with pre-pandemic mental conditions may be therefore better explained by common molecular mechanisms acting at the central level. We have observed that some of the candidate genes of the 17q21.31 inversion locus associated with severity show a strong expression enrichment in the neural system [39]. For example, protein coding genes including *ARL17B*, *STH*, *KANSL1*, *LRRC37A2*, *MAPT*, *NSF*, *PLEKHM1* and *CRHR1* show high expression in brain tissues and pituitary gland. A combined effect of previous conditions and COVID-19 disease may trigger autoimmune and inflammatory processes leading to an exaggerated symptoms progression [13]. While further research is warrant, it is evident that people living with a mental health condition are a vulnerable group that require a special consideration during health emergencies such as the COVID-19 pandemic.

When considering the general population, household interpersonal conflicts were among the strongest factors associated with depression and anxiety after the first outbreak. This effect may have been accentuated by the prolonged lockdown measures, as suggested by the 27.8% of participants reporting difficulties in interpersonal relationships at home during confinement. Interpersonal conflicts include a wide range of behaviors and describe interpersonal dynamics that repeat over time. It is possible that a frequent exposure to these stressful situations underlies the increased risk of depression and anxiety [40, 41]. Financial stressors also showed a relevant association with increased severe depression and anxiety not only among people in low/middle SES but also among those from higher income contexts. Unemployment and inability to pay the bills are a well-acknowledged risk factor for poor mental health that in the context of the pandemic has hit the society as a whole, creating new vulnerabilities and further deepening pre-existing inequalities [6, 42].

Lack of access to outdoor spaces was a main concern during the confinement, because it had previously been linked to changes at the level of the stress response system through direct modulation of affective states, or indirectly through social cohesion and physical activity [43]. Our results support these concerns, and add evidence towards the role of noise as a relevant environmental predictor of both depression and anxiety. We explored this association using a measure of noise annoyance that indicates the subjective emotion related to the experience of noise. This result may in part reflect the fact that noise coming from outside the homes was inescapable during confinement, which could have accentuated the experience of stress [44, 45].

This study has several strengths, including the cohort and population-based design, and sample size. Limitations include the modest response rate mostly due to errors in contact information and an overrepresentation of people with an education level of college degree or higher, as well as aged 45 and above. The prevalence of pre-pandemic diagnosis of mental health disorder was at the lower end of the range expected in the general population according to Spanish registries [46]. This does not affect the validity of the findings but may affect generalizability. The prevalence of severe depression and anxiety in our study are similar to those reported by initial population-based surveys in other settings [15, 47, 48], although the direct comparison between studies should be done cautiously given the methodological differences across them (diagnosis definition and assessment, selection of participants, period of data collection). Our data do not cover young people, who are a particularly vulnerable group and showed a high increase in prevalence of depression and anxiety after the first outbreak [49]. However, people over 40 was the age group with the highest prevalence before the outbreak, and followed younger groups in post-outbreak increased prevalence [50]. The availability of population surveys in the region with detailed information on lifestyle, residence, social class, health conditions and health-related behaviors allowed us to control for differences between our study population and the general population of the wider region and model population prevalence. Finally, the number of participants with COVID-19 disease was small, which could be a limitation of our study. However, the prevalence of COVID-19 in our sample was comparable with that of the underlying population at the time of assessment [51].

In conclusion, we found an increase in prevalence of severe depression and anxiety in relation to the COVID-19 pandemic and subsequent lockdown. Our results raise awareness on the impact of interpersonal conflicts and financial burden on mental wellbeing and highlight the need to address wider environment and societal factors in the management of the emergency. The variation in risk observed among people with pre-pandemic mental health conditions suggest different underlying mechanisms that require tailored interventions.

## Supplementary Information

Below is the link to the electronic supplementary material.

Supplementary file1 (DOCX 52 KB)

## Data Availability

The COVICAT study deidentified participant data and data dictionary will be made available upon request to the corresponding author after approval of a proposal and signed data access agreement.

## References

[1] Holmes EA, O’Connor RC, Perry VH (2020). Multidisciplinary research priorities for the COVID-19 pandemic: a call for action for mental health science. Lancet Psychiatry.

[2] Simon N, Saxe G, Marmar CR (2020). Mental health disorders related to COVID-19 – related deaths. JAMA.

[3] Kessler RC, Bromet EJ (2013). The epidemiology of depression across cultures. Annu Rev Public Health.

[4] Bandelow B, Michaelis S (2015). Epidemiology of anxiety disorders in the 21st century. Dialogues Clin Neurosci.

[5] Allen J, Balfour R, Bell R, Marmot M (2014). Social determinants of mental health. Int Rev Psychiatry.

[6] Chandola T, Kumari M, Booker CL, Benzeval MJ (2020). The mental health impact of COVID-19 and lockdown-related stressors among adults in the UK. Psychol Med.

[7] Kuehner C (2017). Why is depression more common among women than among men?. Lancet Psychiatry.

[8] Fitzpatrick KM, Harris C, Drawve G (2020). Living in the midst of fear: depressive symptomatology among US adults during the COVID-19 pandemic. Depress Anxiety.

[9] Liu L, Ni S-Y, Yan W (2021). Mental and neurological disorders and risk of COVID-19 susceptibility, illness severity and mortality: a systematic review, meta-analysis and call for action. EClinicalMedicine.

[10] Yao H, Chen JH, Xu YF (2020). Patients with mental health disorders in the COVID-19 epidemic. Lancet Psychiatry.

[11] Otte C, Gold SM, Penninx BW (2016). Major depressive disorder. Nat Rev Dis Prim.

[12] Craske MG, Stein MB, Eley TC (2017). Anxiety disorders. Nat Rev Dis Prim.

[13] Moni MA, Lin P-I, Quinn JMW, Eapen V (2021). COVID-19 patient transcriptomic and genomic profiling reveals comorbidity interactions with psychiatric disorders. Transl Psychiatry.

[14] Xia J, Chen S, Li Y (2022). Immune response is key to genetic mechanisms of SARS-CoV-2 infection with psychiatric disorders based on differential gene expression pattern analysis. Front Immunol.

[15] Fancourt D, Steptoe A, Bu F (2021). Trajectories of anxiety and depressive symptoms during enforced isolation due to COVID-19 in England: a longitudinal observational study. Lancet Psychiatry.

[16] Ministerio de Trabajo y Economía Social (2020) Evolución del paro registrado, Madrid

[17] Ministerio de Igualdad (2020) Boletín Estadístico Mensual, Madrid

[18] Obón-Santacana M, Vilardell M, Carreras A (2018). GCAT|Genomes for life: a prospective cohort study of the genomes of Catalonia. BMJ Open.

[19] Zigmond AS, Snaith RP (1983). The hospital anxiety and depression scale. Acta Psychiatr Scand.

[20] Carmen Terol-Cantero M, Cabrera-Perona V, Martín-Aragón M (2015). Revisión de estudios de la Escala de Ansiedad y Depresión Hospitalaria (HAD) en muestras españolas. An Psicol.

[21] Bjelland I, Dahl AA, Haug TT, Neckelmann D (2002). The validity of the hospital anxiety and depression scale: an updated literature review. J Psychosom Res.

[22] Bronfenbrenner U, Ceci SJ (1994). Nature-nurture reconceptualized in developmental perspective: a bioecological model. Psychol Rev.

[23] Karachaliou M, Moncunill G, Espinosa A (2021). Infection induced SARS-CoV-2 seroprevalence and heterogeneity of antibody responses in a general population cohort study in Catalonia Spain. Sci Rep.

[24] Dobaño C, Vidal M, Santano R (2021). Highly sensitive and specific multiplex antibody assays to quantify immunoglobulins M, A, and G against SARS-CoV-2 antigens. J Clin Microbiol.

[25] Dal Grande E, Chittleborough CR, Campostrini S (2015). Health estimates using survey raked-weighting techniques in an Australian population health surveillance system. Am J Epidemiol.

[26] Gobbi S, Płomecka M, Ashraf Z (2020). Worsening of preexisting psychiatric conditions during the COVID-19 pandemic. Front Psychiatry.

[27] Favreau M, Hillert A, Osen B (2021). Psychological consequences and differential impact of the COVID-19 pandemic in patients with mental disorders. Psychiatry Res.

[28] Hao F, Tan W, Jiang L (2020). Do psychiatric patients experience more psychiatric symptoms during COVID-19 pandemic and lockdown? A case-control study with service and research implications for immunopsychiatry. Brain Behav Immun.

[29] Cacioppo JT, Cacioppo S (2018). The growing problem of loneliness. Lancet (London, England).

[30] Burrai J, Roma P, Barchielli B (2020). Psychological and emotional impact of patients living in psychiatric treatment communities during Covid-19 lockdown in Italy. J Clin Med.

[31] Mills EHA, Møller AL, Gnesin F (2020). National all-cause mortality during the COVID-19 pandemic: a Danish registry-based study. Eur J Epidemiol.

[32] Sheridan Rains L, Johnson S, Barnett P (2021). Early impacts of the COVID-19 pandemic on mental health care and on people with mental health conditions: framework synthesis of international experiences and responses. Soc Psychiatry Psychiatr Epidemiol.

[33] Taquet M, Luciano S, Geddes JR, Harrison PJ (2020). Bidirectional associations between COVID-19 and psychiatric disorder: retrospective cohort studies of 62 354 COVID-19 cases in the USA. Lancet Psychiatry.

[34] Lee SW, Yang JM, Moon SY (2020). Association between mental illness and COVID-19 susceptibility and clinical outcomes in South Korea: a nationwide cohort study. Lancet Psychiatry.

[35] Park HY, Song I-A, Lee SH (2021). Prevalence of mental illness among COVID-19 survivors in South Korea: nationwide cohort. BJPsych open.

[36] Taquet M, Dercon Q, Luciano S (2021). Incidence, co-occurrence, and evolution of long-COVID features: a 6-month retrospective cohort study of 273,618 survivors of COVID-19. PLoS Med.

[37] Sass MR, Juul TS, Skov R (2022). SARS-CoV-2 seroprevalence among patients with severe mental illness: a cross-sectional study. PLoS One.

[38] Niemi KME, Karjalainen J, Liao RG (2021). Mapping the human genetic architecture of COVID-19. Nature.

[39] Degenhardt F, Ellinghaus D, Juzenas S (2022). Detailed stratified GWAS analysis for severe COVID-19 in four European populations. medRxiv.

[40] Garcia-Moreno C, Jansen HA, Ellsberg M (2006). Prevalence of intimate partner violence: findings from the WHO multi-country study on women’s health and domestic violence. Lancet.

[41] Coll CVN, Ewerling F, García-Moreno C (2020). Intimate partner violence in 46 low-income and middle-income countries: an appraisal of the most vulnerable groups of women using national health surveys. BMJ Glob Heal.

[42] Wright L, Steptoe A, Fancourt D (2021). Does thinking make it so? Differential associations between adversity worries and experiences and mental health during the COVID-19 pandemic. J Epidemiol Community Health.

[43] Bratman GN, Anderson CB, Berman MG (2019). Nature and mental health: an ecosystem service perspective. Sci Adv.

[44] Orban E, McDonald K, Sutcliffe R (2016). Residential road traffic noise and high depressive symptoms after five years of follow-up: results from the heinz nixdorf recall study. Environ Health Perspect.

[45] Park J, Chung S, Lee J (2017). Noise sensitivity, rather than noise level, predicts the non-auditory effects of noise in community samples: a population-based survey. BMC Public Health.

[46] Ministerio de Sanidad Consumo y Bienestar Social (2017) Informe monográfico de Salud Mental, Madrid

[47] Cénat JM, Noorishad PG, Kokou-Kpolou CK (2021). Prevalence and correlates of depression during the COVID-19 pandemic and the major role of stigmatization in low- and middle-income countries: a multinational cross-sectional study. Psychiatry Res.

[48] Czeisler M, Lane RI, Wiley JF (2021). Follow-up survey of US adult reports of mental health, substance use, and suicidal ideation during the COVID-19 pandemic, september 2020. JAMA Netw open.

[49] Gagné T, Schoon I, McMunn A, Sacker A (2021). Mental distress among young adults in Great Britain: long-term trends and early changes during the COVID-19 pandemic. Soc Psychiatry Psychiatr Epidemiol.

[50] Santomauro DF, Mantilla Herrera AM, Shadid J (2021). Global prevalence and burden of depressive and anxiety disorders in 204 countries and territories in 2020 due to the COVID-19 pandemic. Lancet (London, England).

[51] Equipo COVID-19 (2020) Informe n^o^ 42. Situación de COVID-19 en España, Madrid

