# Supplementary material for: Mental health and COVID-19 in a general population cohort in Spain (COVICAT study)
Source: Soc Psychiatry Psychiatr Epidemiol. 2022 May 28;57(12):2457–68. doi: 10.1007/s00127-022-02303-0 (PMC9142833; doi:10.1007/s00127-022-02303-0)
Supplement: Supplementary file 1 — Supplementary file1 (DOCX 52 KB) [file 127_2022_2303_MOESM1_ESM.docx]

**SUPPLEMENTARY MATERIAL TO THE MANUSCRIPT** **“Mental health and COVID-19 in a general population cohort in Spain (COVICAT study)”**

- - Description of the COVICAT Study cohort
  - S1. Associations between exposures and depression and anxiety by pre-pandemic diagnosis of mental health disorder (Table S1)
  - S2. Associations between exposures and depression and anxiety by gender (Table S2)
  - S3. Associations between exposures and depression and anxiety by age group (Table S3)
  - S4. Associations between exposures and depression and anxiety by SES (Table S4)

Description of the COVICAT Study cohort.

The COVICAT Study sample was sourced from participants of pre-established population-based cohorts plus oversampling with previous cohorts of elder adults and informal worker populations. In the COVICAT Study context, the pre-epidemic data were considered baseline, and the data collected after the first lockdown are considered Wave 1. All participants contacted from the cohort studies had consented in the past to be re-contacted.

The largest proportion of the participants were sourced from the **Genomes for Life (GCAT)** Study (n=8,923). The GCAT cohort study includes middle-aged range (40–65 years old) participants who are residents in Catalonia and recruitment started in 2015. Most participants were enrolled from blood donors invited through the Blood and Tissue Bank (BST), a public agency. Valid contact information (email or telephone) was available for 15,245 participants and of those 8,923 completed an online COVICAT questionnaire or, a small proportion, responded a computer assisted telephone questionnaire (Obon-Santacan et al 2018).

**MCC-Spain** (n=325) includes only the population controls of a population-based multicase-control study launched in 2008 to evaluate the influence of environmental factors in common tumours in 12 provinces in Spain (Castano-Vinyals, et al 2018). Population controls were selected at random from the roster of people registered in Primary Health Care centres within the catchment areas of the hospitals where cases were recruited. For the COVICAT study, we have re-contacted controls living in the provinces of Barcelona and Girona

The **European Community Respiratory Health Study** (n=112) is a population-based study initiated in 1991-1993 to assess the prevalence of asthma and allergic disease in Europe (Burney et al 1994). Young adults (20-44 years old) were randomly-selected from available population-based registers, from 25 countries and 56 centres across Europe. Participants completed a detailed questionnaire at baseline (ECRHS 1), in 2 follow-up surveys (ECRHS 2, ECRHS 3) taken 10 years apart. For the COVICAT study, we have re-contacted participants in the provinces of Barcelona.

Special populations to increase the proportion of older participants and rural area residents (n=55). The **Urban Training** study is a multicentre randomized controlled trial (NCT01897298) on Chronic Obstructive Pulmonary Disease in five Catalan municipalities Arbillaga-Etxarri et al 2018). For the COVICAT study, we re-contacted participants from both intervention arms who, according to medical care records, were known to be alive and cognitively able. The **LeRAgs** is a cross-sectional study including agricultural workers of crops from different climatic conditions in three provinces of Spain. For the COVICAT study, we have contacted participants in the Tarragona province, Catalonia.

References:

1. Obon-Santacana M, Vilardell M, Carreras A, et al. GCAT|Genomes for life: a prospective cohort study of the genomes of Catalonia. BMJ Open 2018;8:e018324. doi:10.1136/bmjopen-2017-018324
2. Castaño-Vinyals G, Aragonés N, Pérez-Gómez B, Martín V, Llorca J, Moreno V, Altzibar JM, Ardanaz E, de Sanjosé S, Jiménez-Moleón JJ, Tardón A, Alguacil J, Peiró R, Marcos-Gragera R, Navarro C, Pollán M, Kogevinas M; MCC-Spain Study Group. Population-based multicase-control study in common tumors in Spain (MCC-Spain): rationale and study design. Gac Sanit. 2015 Jul-Aug;29(4):308-15. doi: 10.1016/j.gaceta.2014.12.003. Epub 2015 Jan 19. Erratum in: Gac Sanit. 2018 Sep - Oct;32(5):501. PMID: 25613680.
3. Arbillaga-Etxarri A, Gimeno-Santos E, Barberan-Garcia A, Balcells E, Benet M, Borrell E, Celorrio N, Delgado A, Jané C, Marin A, Martín-Cantera C, Monteagudo M, Montellà N, Muñoz L, Ortega P, Rodríguez DA, Rodríguez-Roisin R, Simonet P, Torán-Monserrat P, Torrent-Pallicer J, Vall-Casas P, Vilaró J, Garcia-Aymerich J. Long-term efficacy and effectiveness of a behavioural and community-based exercise intervention (Urban Training) to increase physical activity in patients with COPD: a randomised controlled trial. Eur Respir J. 2018 Oct 18;52(4):1800063. doi: 10.1183/13993003.00063-2018. PMID: 30166322; PMCID: PMC6203405.
4. Burney PG, Luczynska C, Chinn S, Jarvis D. The European Community Respiratory Health Survey. Eur Respir J. 1994 May;7(5):954-60. doi: 10.1183/09031936.94.07050954.

**Supplementary Table S1. Associations between exposures and severe depression and anxiety by pre-pandemic diagnosis of mental health (MH) disorder.** Table shows the results of the log-binomial/Poisson regression models. The reference value for each regression model was no depression and no anxiety, respectively.

|  | **Depression** | |  | **Anxiety** | |  |
| --- | --- | --- | --- | --- | --- | --- |
|  | **Pre-pandemic MH Diagnosis** | **No pre-pandemic MH diagnosis** |  | **Pre-pandemic MH Diagnosis** | **No pre-pandemic MH diagnosis** |  |
|  | RR (95% CI) | RR (95% CI) | p-value** | RR (95% CI) | RR (95% CI) | p-value** |
| **Household conditions** |  |  |  |  |  |  |
| Living alone | 1.59 (1.17 - 2.15) | 1.25 (0.97 - 1.61) | 0.24 | 1.10 (0.84 - 1.43) | 0.88 (0.73 - 1.07) | 0.20 |
| High media exposure (*) | 1.34 (1.03 - 1.76) | 1.65 (1.35 - 2.01) | 0.23 | 1.35 (1.12 - 1.63) | 1.67 (1.47 - 1.89) | 0.07 |
| Interpersonal conflicts | 1.47 (1.14 - 1.89) | 2.56 (2.13 - 3.08) | 0.00 | 1.42 (1.19 - 1.70) | 2.13 (1.89 - 2.40) | 0.00 |
| Caregiving of children | 0.95 (0.73 - 1.26) | 1.29 (1.04 - 1.60) | 0.08 | 0.98 (0.81 - 1.18) | 1.20 (1.04 - 1.38) | 0.08 |
| **Financial strain** |  |  |  |  |  |  |
| Currently unemployed | 1.45 (1.08 - 1.95) | 1.98 (1.57 - 2.51) | 0.10 | 1.26 (1.01 - 1.58) | 1.36 (1.15 - 1.62) | 0.59 |
| Struggle to pay rent/food | 1.73 (1.34 - 2.23) | 2.24 (1.77 - 2.85) | 0.14 | 1.58 (1.32 - 1.88) | 1.86 (1.58 - 2.18) | 0.18 |
| **Wider environment** |  |  |  |  |  |  |
| Rare/no access to outdoor spaces (*) | 1.09 (0.84 - 1.43) | 1.67 (1.37 - 2.04) | 0.01 | 1.02 (0.83 - 1.24) | 1.32 (1.16 - 1.51) | 0.03 |
| Noise annoyance | 1.16 (0.90 - 1.50) | 1.54 (1.28 - 1.86) | 0.08 | 1.32 (1.10 - 1.58) | 1.44 (1.27 - 1.63) | 0.43 |
| **COVID-19 (***)** |  |  |  |  |  |  |
| All cases | 1.13 (0.73 - 1.74) | 1.83 (1.32 - 2.55) | 0.08 | 1.11 (0.82 - 1.49) | 1.65 (1.33 - 2.04) | 0.03 |

*Rows represent log-binominal regression models adjusted for age, gender, education level, days passed since end of stay-at-home order, positive COVID-19 diagnosis, and type of interview. Poisson regression models with robust standard errors were applied when convergence was not achieved. Sample sizes were N=9,515 for all models except those marked with (*), which were N=9,430 due to the omission of these questions in the telephone-based interviews.*

*** p-value for the interaction term (Wald test).*

**** Model for COVID-19 adjusted as above except for positive COVID-19 diagnosis.*

**Supplementary Table S2. Associations between exposures and depression and anxiety by gender.** Table shows the results of the log-binomial/Poisson regression models. The reference value for each regression model was no depression and no anxiety, respectively.

|  | **Depression** | |  | **Anxiety** | |  |
| --- | --- | --- | --- | --- | --- | --- |
|  | **Male** | **Female** |  | **Male** | **Female** |  |
|  | RR (95% CI) | RR (95% CI) | p-value** | RR (95% CI) | RR (95% CI) | p-value** |
| **Household conditions** |  |  |  |  |  |  |
| Living alone | 1.52 (1.06 - 2.19) | 1.28 (1.01 - 1.63) | 0.45 | 1.06 (0.77 - 1.45) | 0.90 (0.75 - 1.08) | 0.39 |
| High media exposure (*) | 1.06 (0.77 - 1.45) | 0.90 (0.75 - 1.08) | 0.49 | 1.68 (1.34 - 2.10) | 1.57 (1.39 - 1.77) | 0.60 |
| Interpersonal conflicts | 2.09 (1.54 - 2.82) | 2.27 (1.90 - 2.71) | 0.63 | 2.34 (1.89 - 2.90) | 2.34 (1.89 - 2.90) | 0.06 |
| Caregiving of children | 0.66 (0.31 - 1.42) | 1.23 (1.02 - 1.49) | 0.12 | 0.97 (0.60 - 1.59) | 1.15 (1.02 - 1.31) | 0.52 |
| **Financial strain** |  |  |  |  |  |  |
| Currently unemployed | 2.29 (1.62 - 3.24) | 1.66 (1.32 - 2.08) | 0.12 | 1.56 (1.16 - 2.08) | 1.26 (1.07 - 1.49) | 0.22 |
| Struggle to pay rent/food | 2.63 (1.87 - 3.72) | 1.86 (1.50 - 2.30) | 0.09 | 2.39 (1.86 - 3.08) | 1.59 (1.37 - 1.84) | 0.01 |
| **Wider environment** |  |  |  |  |  |  |
| Rare/no access to outdoor spaces (*) | 1.38 (1.00 - 1.89) | 1.54 (1.27 - 1.86) | 0.55 | 1.26 (1.00 - 1.59) | 1.24 (1.09 - 1.41) | 0.91 |
| Noise annoyance | 1.50 (0.80 - 2.79) | 1.58 (1.17 - 2.14) | 0.03 | 1.69 (1.36 - 2.10) | 1.33 (1.18 - 1.50) | 0.06 |
| **COVID-19 (***)** |  |  |  |  |  |  |
| All cases | 1.50 (0.80 - 2.79) | 1.58 (1.17 - 2.14) | 0.88 | 2.02 (1.38 - 2.95) | 1.36 (1.11 - 1.67) | 0.07 |

*Rows represent log-binominal regression models adjusted for age, education level, days passed since end of stay-at-home order, positive COVID-19 diagnosis, , pre-pandemic diagnosis of mental health disorder and type of interview. Poisson regression models with robust standard errors were applied when convergence was not achieved. Sample sizes were N=9,515 for all models except those marked with (*), which were N=9,430 due to the omission of these questions in the telephone-based interviews.*

*** p-value for the interaction term (Wald test).*

**** Model for COVID-19 adjusted as above except for positive COVID-19 diagnosis.*

**Supplementary Table S3. Associations between exposures and severe depression and anxiety by age group.** Table shows the results of the log-binomial/Poisson regression models. The reference value for each regression model was no depression and no anxiety, respectively.

|  | **Depression** | | |  | **Anxiety** | |  |  |
| --- | --- | --- | --- | --- | --- | --- | --- | --- |
|  | **49 y/o and below** | **50 to 59 y/o** | **60 y/o and above** |  | **49 y/o and below** | **50 to 59 y/o** | **60 y/o and above** |  |
|  | RR (95% CI) | RR (95% CI) | RR (95% CI) | p-value** | RR (95% CI) | RR (95% CI) | RR (95% CI) | p-value** |
| **Household conditions** |  |  |  |  |  |  |  |  |
| Living alone | 1.61 (1.13 - 2.29) | 1.20 (0.89 - 1.61) | 1.34 (0.87 - 2.06) | 0.46 | 1.03 (0.79 - 1.35) | 0.91 (0.72 - 1.15) | 0.88 (0.63 - 1.24) | 0.72 |
| High media exposure (*) | 1.60 (1.19 - 2.13) | 1.58 (1.26 - 1.98) | 1.43 (0.94 - 2.16) | 0.90 | 1.46 (1.21 - 1.77) | 1.62 (1.39 - 1.88) | 1.83 (1.37 - 2.43) | 0.42 |
| Interpersonal conflicts | 2.11 (1.64 - 2.71) | 2.26 (1.82 - 2.80) | 2.39 (1.60 - 3.58) | 0.85 | 1.96 (1.65 - 2.31) | 1.83 (1.58 - 2.12) | 2.59 (1.95 - 3.44) | 0.10 |
| Caregiving of children | 1.10 (0.84 - 1.45) | 1.19 (0.93 - 1.51) | 1.52 (0.86 - 2.68) | 0.60 | 1.13 (0.95 - 1.35) | 1.07 (0.91 - 1.27) | 1.66 (1.13 - 2.43) | 0.12 |
| **Financial strain** |  |  |  |  |  |  |  |  |
| Currently unemployed | 1.76 (1.31 - 2.37) | 1.69 (1.28 - 2.23) | 2.71 (1.57 - 4.68) | 0.30 | 1.29 (1.04 - 1.61) | 1.31 (1.07 - 1.61) | 1.61 (1.02 - 2.52) | 0.68 |
| Struggle to pay rent/food | 2.21 (1.66 - 2.94) | 2.07 (1.60 - 2.67) | 1.43 (0.77 - 2.65) | 0.45 | 1.63 (1.32 - 2.00) | 1.85 (1.55 - 2.20) | 1.93 (1.30 - 2.85) | 0.58 |
| **Wider environment** |  |  |  |  |  |  |  |  |
| Rare/no access to outdoor spaces (*) | 1.26 (0.97 - 1.66) | 1.57 (1.24 - 1.97) | 1.96 (1.27 - 3.00) | 0.20 | 1.19 (0.99 - 1.43) | 1.23 (1.05 - 1.45) | 1.47 (1.07 - 2.03) | 0.53 |
| Noise annoyance | 1.37 (1.06 - 1.77) | 1.45 (1.16 - 1.80) | 1.58 (1.04 - 2.41) | 0.83 | 1.35 (1.14 - 1.60) | 1.32 (1.14 - 1.53) | 2.07 (1.56 - 2.75) | 0.02 |
| **COVID-19 (***)** |  |  |  |  |  |  |  |  |
| All cases | 1.68 (1.05 - 2.67) | 1.39 (0.95 - 2.04) | 1.98 (1.00 - 3.92) | 0.64 | 1.47 (1.07 - 2.00) | 1.38 (1.08 - 0.00) | 2.07 (1.28 - 0.00) | 0.35 |

*Rows represent log-binominal regression models adjusted for gender, education level, days passed since end of stay-at-home order, positive COVID-19 diagnosis, pre-pandemic diagnosis of mental health disorder and type of interview. Poisson regression models with robust standard errors were applied when convergence was not achieved. Sample sizes were N=9,515 for all models except those marked with (*), which were N=9,430 due to the omission of these questions in the telephone-based interviews.*

*** p-value for the interaction term (Wald test).*

**** Model for COVID-19 adjusted as above except for positive COVID-19 diagnosis.*

**Supplementary Table S4. Associations between exposures and depression and anxiety by socioeconomic (SES) group.** Table shows the results of the log-binomial/Poisson regression models. The reference value for each regression model was no depression and no anxiety, respectively.

|  | **Depression** | |  | **Anxiety** | |  |
| --- | --- | --- | --- | --- | --- | --- |
|  | **Low/Middle SES** | **High SES** |  | **Low/Middle SES** | **High SES** |  |
|  | RR (95% CI) | RR (95% CI) | p-value** | RR (95% CI) | RR (95% CI) | p-value** |
| **Household conditions** |  |  |  |  |  |  |
| Living alone | 1.48 (1.15 - 1.89) | 1.16 (0.83 - 1.63) | 0.26 | 1.03 (0.85 - 1.25) | 0.81 (0.62 - 1.06) | 0.15 |
| High media exposure (*) | 1.64 (1.33 - 2.01) | 1.44 (1.10 - 1.88) | 0.45 | 1.83 (1.60 - 2.09) | 1.27 (1.06 - 1.54) | 0.00 |
| Interpersonal conflicts | 2.17 (1.78 - 2.63) | 2.32 (1.81 - 2.98) | 0.67 | 1.83 (1.60 - 2.08) | 2.22 (1.87 - 2.63) | 0.08 |
| Caregiving of children | 1.07 (0.86 - 1.34) | 1.37 (1.04 - 1.81) | 0.16 | 1.01 (0.87 - 1.17) | 1.37 (1.13 - 1.66) | 0.01 |
| **Financial strain** |  |  |  |  |  |  |
| Currently unemployed | 1.93 (1.54 - 2.41) | 1.47 (0.96 - 2.23) | 0.26 | 1.42 (1.21 - 1.66) | 1.05 (0.74 - 1.49) | 0.12 |
| Struggle to pay rent/food | 2.00 (1.62 - 2.48) | 2.28 (1.60 - 3.25) | 0.54 | 1.83 (1.58 - 2.12) | 1.58 (1.20 - 2.09) | 0.37 |
| **Wider environment** |  |  |  |  |  |  |
| Rare/no access to outdoor spaces (*) | 1.46 (1.19 - 1.79) | 1.54 (1.18 - 2.02) | 0.74 | 1.22 (1.06 - 1.41) | 1.28 (1.06 - 1.55) | 0.69 |
| Noise annoyance | 1.62 (1.33 - 1.97) | 1.18 (0.90 - 1.54) | 0.06 | 1.47 (1.29 - 1.67) | 1.34 (1.12 - 1.60) | 0.42 |
| **COVID-19 (***)** |  |  |  |  |  |  |
| All cases | 1.55 (1.11 - 2.17) | 1.56 (0.99 - 2.48) | 0.98 | 1.60 (1.28 - 1.99) | 1.28 (0.92 - 1.80) | 0.29 |

*Rows represent univariate regression models adjusted for age, gender, days passed since end of stay-at-home order, positive COVID-19 diagnosis, pre-pandemic diagnosis of mental health disorder, and type of interview. Poisson regression models with robust standard errors were applied when convergence was not achieved. Sample sizes were N=9,515 for all models except those marked with (*), which were N=9,430 due to the omission of these questions in the telephone-based interviews.*

*** p-value for the interaction term (Wald test).*

**** Model for COVID-19 adjusted as above except for positive COVID-19 diagnosis.*
